# Whole-Genome Sequence of the Microcin E492-Producing Strain *Klebsiella pneumoniae* RYC492

**DOI:** 10.1128/genomeA.00178-13

**Published:** 2013-05-09

**Authors:** Andrés Marcoleta, Sergio Gutiérrez-Cortez, Daniel Maturana, Octavio Monasterio, Rosalba Lagos

**Affiliations:** Departamento de Biología, Facultad de Ciencias, Universidad de Chile, Santiago, Chile

## Abstract

Here, we report the draft genome sequence of the Gram-negative strain *Klebsiella pneumoniae* RYC492, which produces the amyloid-forming and antibacterial peptide microcin E492. The sequenced genome consists of a 5,095,761-bp assembled open chromosome where the gene cluster for microcin production is located in a putative 31-kb genomic island flanked by sequence repeats and containing a putative integrase-coding gene.

## GENOME ANNOUNCEMENT

Microcin E492 (MccE492) is a bacteriocin produced by *Klebsiella pneumoniae* RYC492, a Gram-negative strain (1) that is active against members of the *Enterobacteriaceae* family (2). MccE492 also is toxic and induces apoptosis in malignant human cell lines (3); it aggregates into amyloid-like fibers, thereby modulating its antibacterial properties (4, 5), and its C terminus can be postranslationally modified with salmochelin-like molecules (6, 7). The genetic determinants for MccE492 production are located in the chromosome of *K. pneumoniae* RYC492, clustered in a ~13,000-bp segment, which was cloned and studied in *Escherichia coli* (8, 9). Although microcin production is encoded in this segment, the genetic context in the original strain has not been described. To further characterize the MccE492 gene cluster, we sequenced and annotated the genome of the original microcin producer *K. pneumoniae* RYC492.

The genomic DNA of *K. pneumoniae* RYC492 was obtained using the AxyPrep bacterial genomic DNA miniprep kit (Axygen Biosciences), and its sequence was determined at OMICS Solutions (Santiago, Chile) using an Ion Torrent personal genome machine (PGM), according to the manufacturer’s instructions (10). A total of 2,601,098 reads (278,340,131 bp; mean length, 107 bp) were generated. The raw sequences were assembled with CLC Genomics Workbench 5.5.1 (CLC bio, Denmark) using six different *K. pneumoniae* reference genome sequences (accession no. NC018522, NC011283, NC012731, NC009648, NC017540, and NC016845). The NC018522 sequence, from *K. pneumoniae* strain 1084, was selected to assemble 82.1% of the reads, which were completed with segments (3.37% of the reads) identified with Mauve (11) and Artemis (12) that did not map to the NC018522 sequence but were present in the other reference genome sequences mentioned above. This multireference assembly resulted in the draft sequence of the *K. pneumoniae* RYC492 chromosome (46-fold coverage), with a length of 5,095,761 bp and a G+C content of 57.89%. The reads not included in the chromosome assembly were used to perform a *de novo* assembly, generating 158 contigs that contained a total of 288,021 bp. Both the chromosome and contigs were annotated using the NCBI Prokaryotic Genomes Automatic Annotation Pipeline, revealing a total of 5,095 protein-coding genes, 77 tRNAs, 6 copies of 16S rRNA, 8 copies of 23S rRNA, and 9 copies of 5S rRNA. The MccE492 gene cluster was found to be included in a putative genomic island of approximately 31 kb (G+C content, 48.34%), inserted into an Asn-tRNA site, flanked by repeated sequences and containing an integrase-coding gene.

Among the nonchromosomal genes, we found two distinct replication systems (RepA and ColE1), suggesting the existence of compatible plasmids in the RYC492 strain, as reported previously (13). The draft genome presented here will help in making inferences about the evolutive relationship between the MccE942 gene cluster and genes in other microcin-producing bacteria from the same family, and it will contribute to a better understanding of microcin production and regulation in the original strain.

### Nucleotide sequence accession numbers.

This *K. pneumoniae* RYC492 Whole-Genome Shotgun project has been deposited at DDBJ/EMBL/GenBank under the accession no. APGM00000000. The version described in this paper is the first version, accession no. APGM01000000.
